# The Effects of Antimicrobial Photodynamic Therapy Used to Sterilize Carious Dentin on Rat Dental Pulp Tissue

**DOI:** 10.3390/dj11120283

**Published:** 2023-12-06

**Authors:** Tenyu Takahashi, Fumiaki Sato, Koichi Shinkai

**Affiliations:** 1 Advanced Operative Dentistry-Endodontics, The Nippon Dental University Graduate School of Life Dentistry at Niigata, 1-8 Hamaura-cho, Chuo-ku, Niigata 951-8580, Japan; takaten@ngt.ndu.ac.jp; 2 Department of Operative Dentistry, The Nippon Dental University School of Life Dentistry at Niigata, 1-8 Hamaura-cho, Chuo-ku, Niigata 951-8580, Japan; f.sato@ngt.ndu.ac.jp

**Keywords:** antimicrobial photodynamic therapy, methylene blue, brilliant blue, pulp, semiconductor laser

## Abstract

Antimicrobial photodynamic therapy (aPDT) used to sterilize carious dentin may irritate pulp tissues because of tissue-penetrating laser and singlet oxygen generation. This study aimed to assess the effects of aPDT on rat pulp tissues. A cavity formed in a rat maxillary first molar was treated with aPDT. The combined photosensitizer and laser irradiation conditions in the aPDT groups were as follows: methylene blue and 100 mW for 60 s, brilliant blue (BB) and 100 mW for 60 s, BB and 50 mW for 120 s, and BB and 200 mW for 30 s. Each cavity was treated with an all-in-one adhesive and filled with flowable resin. aPDT was not applied for the control. In each group, the rats were sacrificed on postoperative days 1 and 14, and thin sections of the treated teeth were prepared. Pulp tissue disorganization (PTD), inflammatory cell infiltration (ICI), and tertiary dentin formation (TDF) were evaluated. At 1-day evaluation, there were significant differences between the aPDT group and controls with respect to PTD and ICI (*p* < 0.01); 14 days later, almost all specimens showed tertiary dentin formation. The application of aPDT caused reversible damage to the rat pulp, while in the long term, healing occurred with the formation of tertiary dentin.

## 1. Introduction

Laser devices of various wavelengths are clinically applied in dentistry. Laser devices can be divided into two types according to wavelength: surface-absorbing and tissue-penetrating lasers. Surface-absorbing lasers include carbon dioxide lasers (10.6 µm) and Er:YAG lasers (2.94 µm), whereas tissue-transmitting lasers include Nd:YAG lasers (1.06 µm) and semiconductor lasers (0.6–0.9 µm). The applications of dental lasers for various dental diseases depend on the wavelength, and the laser device should be selected according to the contents of dental treatment [1].

Tissue-penetrating semiconductor lasers are used for antibacterial photodynamic therapy (aPDT) [2]. aPDT uses singlet oxygen produced by irradiating a photosensitizer (PS) with a laser of appropriate wavelength to kill bacteria and is mainly used for endodontic treatment, periodontitis, and periodontitis of dental implants [3,4,5,6,7,8]. According to these previous studies, local disinfection of infected root canals and periodontal pockets with small doses of antimicrobial agents may lead to the emergence of resistant bacteria, whereas the application of aPDT may prevent the emergence of resistant bacteria. Although localized disinfection of infected dentin with antimicrobial agents is also considered, the emergence of resistant bacteria is a concern as well. The bactericidal effect on cariogenic bacteria has also been investigated for the application of aPDT to caries treatment in the clinic. Previous studies using agar medium and dentin plates have confirmed the bactericidal effect of aPDT on cariogenic bacteria such as *Streptococcus mutans* and *Lactobacillus* [9,10,11,12]. Nagai et al. reported that aPDT combining a photosensitizer and semiconductor laser irradiation was applied to bovine dentin fragment infected with *Streptococcus mutans*, and aPDT combining a semiconductor laser with a wavelength of 650 nm and methylene blue showed bactericidal effects against *Streptococcus mutans* [9]. Yoshii et al. found that aPDT combining same wavelength semiconductor laser and brilliant blue was bactericidal for *Lactobacillus acidophilus* [10]. Ahrari et al. reported that aPDT combining a semiconductor laser with a wavelength of 810 nm and indocyanine green was bactericidal for *Lactobacillus acidophilus*. Thus, semiconductor lasers with various wavelengths were utilized for aPDT [12].

The indirect pulp capping (IPC) method treats deep dental caries near the pulp to avoid pulp exposure. In the IPC method, the deepest layer of the carious dentin is incidentally left in place, an IPC material such as calcium hydroxide is placed over the remaining carious dentin to promote restorative dentin formation, and the cavity is temporally filled with dental cement or resin composite. After several months of observing the IPC-treated tooth, the temporary filling in the cavity and the remaining carious dentin are completely removed and finally restored [13,14,15,16]. However, IPC is complex, requiring a long observation period and re-entry. Therefore, developing new treatments that simplify the treatment of deep caries would be clinically efficient and beneficial. Applying aPDT in caries treatment can sterilize the remaining carious dentin and allow for the immediate final restoration of deep carious dentin, making it a simple treatment method for deep caries.

aPDT caries treatment uses a tissue-permeable semiconductor laser beam and singlet oxygen to sterilize deep carious dentin, which may damage pulp tissues adjacent to the deep caries. A previous study has reported the effects of laser irradiation with semiconductor laser devices on dental pulp [17]. This study reported that the application of laser to direct pulp capping resulted in the removal of contaminants, hemostasis, and accelerated wound healing at the exposed pulp surface. Therefore, aPDT in combination with laser may not only sterilize infected dentin but may also reduce pulpal irritation reactions caused by bacterial metabolites. However, the effects of aPDT on the pulp remain unclear. Thus, this study investigated the effects of aPDT on rat dental pulp using a combination of two types of PS and three types of laser irradiation conditions. The null hypothesis was that aPDT would not damage the rat dental pulp, regardless of PS and laser irradiation conditions.

## 2. Materials and Methods

### 2.1. Animals

This study was approved by the Laboratory Animal Committee of The Nippon Dental University School of Life Dentistry at Niigata (Approval No. 226). Male Sprague–Dawley rats (8 weeks old, 300–400 g) were used for this study and fed water and solid food (CRF-1, Oriental Yeast Industry Co., Ltd., Tokyo, Japan) for 1–3 weeks. Rat maxillary first molars were used, and teeth with large cavities, pulp exposures, and suspected fractures were excluded. Thirty-two rats were examined in this study.

### 2.2. Materials and Experimental Groups

The materials used in this study and the experimental groups are shown in Table 1; Table 2, respectively. BeautiBond Xtreme (Shofu Inc., Kyoto, Japan), a one-step self-etching system, and Beautifil Flow Plus X F00 (Shofu Inc.), a flowable resin composite, were selected. The PSs used were brilliant blue (BB) and methylene blue (MB). The semiconductor laser device was the P2 Dental Laser System (Pioon Laser Technology Co., Ltd., Wuhan, China) with a wavelength of 650 nm.

Five experimental groups were set up, including a control, using a combination of three laser irradiation conditions and two types of PS.

### 2.3. Specimen Preparation

Rats were administered a mixture of medetomidine (Domitor^®^, Nippon Zenyaku Kogyo Co., Fukushima, Japan), midazolam (Dormicum^®^, Astellas Pharma, Tokyo, Japan), and butorphanol (Vetorphale^®^, Meiji Seika Pharma Co., Tokyo, Japan) intraperitoneally at a dose ratio of 0.15:2.0:2.5 mg/kg. The rats were fixed to the operating table, and their mouths were held open with a rat mouth opener and first disinfected with 3% H_2_O_2_ and diluted iodine tincture.

A diamond point (FG#MI-F06RL, Lot#081730, Shofu Inc., Kyoto, Japan) was attached to the hand piece, and a cavity (approximately 1.0 mm in diameter and 0.6 mm deep) was prepared with the diamond point on the mesial proximal surface of the maxillary first molar under water irrigation. The cavity was prepared under a magnified view (25.6×) using a dental microscope (Bright Vision, Pentron Japan Inc., Tokyo, Japan).

In each experimental group, except for the control group, each PS was applied to the cavity, and then the laser was irradiated according to the respective irradiation conditions. After an ascorbic acid solution was applied to the cavity to remove residual active oxygen, BeautiBond Xtreme (Shofu Inc.) was applied to the cavities, left for 20 s, and then air-blown to make the bonding layer thin, followed by light irradiation for 5 s using an LED lamp (Pencure, Morita Manufacturing Co., Ltd., Kyoto, Japan). After tooth surface treatment, the cavity was filled with Beautifil Flow Plus X F00 (Shofu Inc.) and photo-polymerized for 10 s. The control cavities were treated with the same adhesive immediately after cavity preparation and filled with the same flowable resin composite.

### 2.4. Perfusion Fixation

On postoperative day 1 or 14, rats were sacrificed by intraperitoneal overdose of a mixed anesthetic of medetomidine, midazolam, and butorphanol, and 4% paraformaldehyde phosphate buffer (PFA) of pH 7.4 was perfused through the left ventricle to fix the dental pulp tissue. The maxillary bones containing the treated teeth were then carefully extracted, and the extracts were immersed overnight in 4% PFA at 4 °C to further fix the pulp tissues.

### 2.5. Preparation of Serial Thin Sections

After removing the excess tissue from the surface of the extracts, the extracts were immersed in a 10% EDTA solution (Decalcifying Soln. B, pH 7.5, FUJIFILM Wako Pure Chemical Co., Osaka, Japan) for 5 weeks at room temperature to decalcify them. After decalcification, flowable resins were carefully removed from the cavities and washed under running water for 3 h. Then, the specimens were dehydrated with ascending grades of ethanol, dealcoholized with xylene, and embedded in paraffin. A sliding microtome was used to prepare 6 µm thick serial thin sections, and the sections were stained with hematoxylin and eosin.

### 2.6. Histological Evaluation

The stained thin sections were observed using an optical microscope (Eclipse E1000, Nikon, Tokyo, Japan) at 40–200× magnification to evaluate three items: pulp tissue disorganization (PTD), inflammatory cell infiltration (ICI), and tertiary dentin formation (TDF). The evaluation criteria for each item are as follows:

▪ PTD

None: normal or nearly normal tissue morphology;

Mild: destruction of odontoblast layer (normal deep pulp morphology);

Moderate: loss of general tissue morphology;

Severe: pulpal necrosis of more than 1/3 of the crown side.

▪ ICI

None: no inflammatory cells or very few inflammatory cells;

Mild: mild acute or chronic inflammatory cell infiltration;

Moderate: moderate inflammatory cell infiltration extending beyond 1/3 of the pulp;

Severe: severe pulp necrosis or pulp loss extending beyond 1/2 of the pulp.

▪ TDF

None: no tertiary dentin formation;

Mild: negligible tertiary dentin formation;

Moderate: moderate tertiary dentin formation;

Severe: high tertiary dentin formation.

### 2.7. Immunostaining and Observation

Thin sections were deparaffinized with xylene and then de-xylene with ethanol. Then, they were washed briefly with tap water and phosphate-buffered saline (PBS: pH 7.4).

For immunohistochemical staining, endogenous peroxidase was blocked with 3% hydrogen peroxide water, immersed in 10% normal goat serum (Lot #H2208A, Nichirei Biosciences Inc., Tokyo, Japan), and incubated for 10 min at room temperature. Polyclonal anti-heat shock protein 27 (HSP27; S78/82, Lot #CN89330, 1:200, Bioworld Technology Inc., St Louis Park, MN, USA) was used as the primary antibody, incubated at 4 °C for 12 h, and then washed twice in PBS for 10 min each. Histofine Simple Stain rat MAX-PO (R) (Lot #H2201A, Nichirei Biosciences Inc., Tokyo, Japan) was used as a secondary antibody, incubated at room temperature for 30 min, and washed twice with PBS for 10 min. 3,3′-Diaminobenzidine tetrahydrochloride (DAB; Histofine Simple Stain DAB Solution, Lot #H2209A, Nichirei Biosciences Inc., Tokyo, Japan) peroxidase activation was used to detect antibody-localized antigens for 10 min at room temperature. Stained thin sections were observed under an optical microscope (Eclipse E1000).

For immunofluorescence staining, nonspecific binding sites were blocked with 10% normal goat serum for 30 min at room temperature, followed by incubation with monoclonal anti-differentiation cluster 146 antibody (CD146; P1H12, Lot #4162-2XP220909, 1:50, Novus biologicals LLc., Centennial, CO, USA) as a primary antibody for 24 h at 4 °C and then washed twice with PBS for 10 min. Then, Alexa Fluor 488-conjugated goat anti-mouse IgG (H + L; Lot #GR3442384-1, 1:100, Abcam Inc., Cambridge, UK) was used as a secondary antibody, exposed for 2 h at room temperature, and washed twice with PBS for 10 min. Stained thin sections were observed under an all-in-one fluorescence microscope (BZ-X710, KEYENCE, Osaka, Japan).

### 2.8. Measurement of the Remaining Dentin Thickness

The thickness of each section’s remaining dentin (distance from the deepest part of the cavity to the pulp chamber) was measured using a stereomicroscope (Nikon Measurescope Model II; Nikon Corporation, Tokyo, Japan). The shortest distance in the measurements was recorded as the specimen’s remaining dentinal thickness.

### 2.9. Statistical Analysis

The remaining dentinal thickness among the experimental groups was compared by Kruskal–Wallis and Steel–Dwass tests at a 5% significance level. Histopathological evaluation data for each observation item among experimental groups at each observation period were statistically analyzed using Kruskal–Wallis and Steel–Dwass tests, and those between the observation terms (on postoperative days 1 and 14) were analyzed using the Mann–Whitney U test at a 5% significance level.

## 3. Results

### 3.1. Thinnest Diameter of the Remaining Dentin

Table 3 shows the mean, standard deviation, median, and minimum/maximum values of the residual dentin thinnest diameter specimens in each experimental group by the observation period. Statistical analysis showed no significant differences between the experimental groups for any observation period (*p* > 0.05).

### 3.2. Results of the Histopathological Evaluation

**Figure 1:**
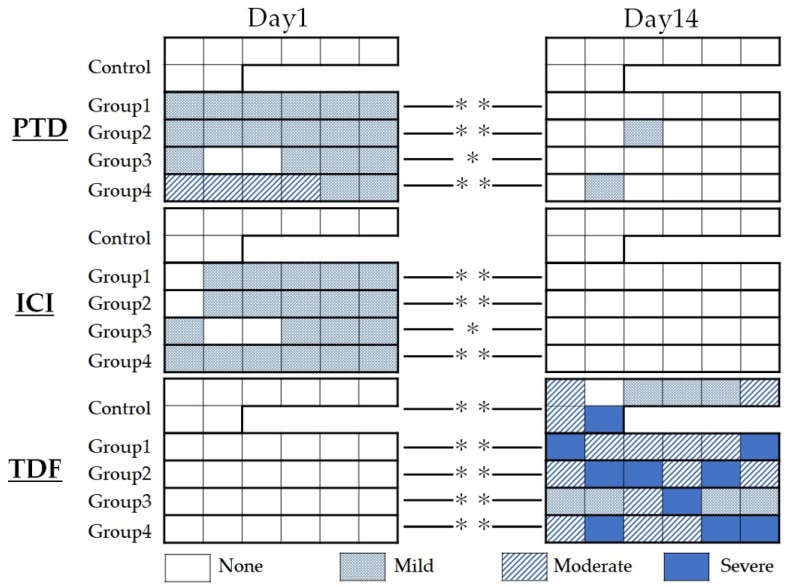
Summary of histopathological evaluation at postoperative days 1 and 14. Pulp tissue disorganization (PTD), inflammatory cell infiltration (ICI), and tertiary dentin formation (TDF) were evaluated. * *p* < 0.05, ** *p* < 0.01.

Figure 1 shows the summary of the results of the histopathological evaluation.

On PTD evaluation, most specimens in the aPDT groups, except for group 4, were rated “mild” because most showed loss of the odontoblastic layer or odontoblast malalignment within the odontoblastic layer. All control specimens were rated “none” because there was no change in the odontoblastic layer on postoperative day 1. In group 4, four of six cases were rated moderate. On postoperative day 14, most specimens in all experimental groups showed a normal organization structure of the odontoblastic layer and were rated as “none.” The results of the Kruskal–Wallis test showed a significant difference between the control and the respective aPDT group on postoperative day 1 (*p* < 0.01); however, no significant difference was observed among all experimental groups on postoperative day 14 (*p* > 0.05). 

On ICI evaluation, all aPDT groups were rated “mild” because most specimens in each aPDT group showed mild inflammatory round cell infiltration, whereas all control specimens showed no round cell infiltration and were all rated “none” on postoperative day 1. On the contrary, on postoperative day 14, all specimens were rated “none” because no inflammatory round-cell infiltration was observed. The results of the Kruskal–Wallis test showed a significant difference between the control and the respective aPDT group on postoperative day 1 (*p* < 0.01); however, no significant differences were noted among all experimental groups on postoperatively day 14 (*p* > 0.05). 

On TDF evaluation, all specimens were rated “none” because no postoperative tertiary dentin formation was observed on day 1. Although the degree of formation varied in the same experimental group, most specimens showed tertiary dentin formation, except for one control specimen on postoperative day 14. The results of the Kruskal–Wallis test showed no significant differences among aPDT groups on postoperative day 14 (*p* > 0.05). 

Statistical comparisons using the Mann–Whitney U test on all evaluation items showed a significant difference between the two observation terms in all aPDT groups (*p* < 0.05).

### 3.3. Histopathological Observation

**Figure 2:**
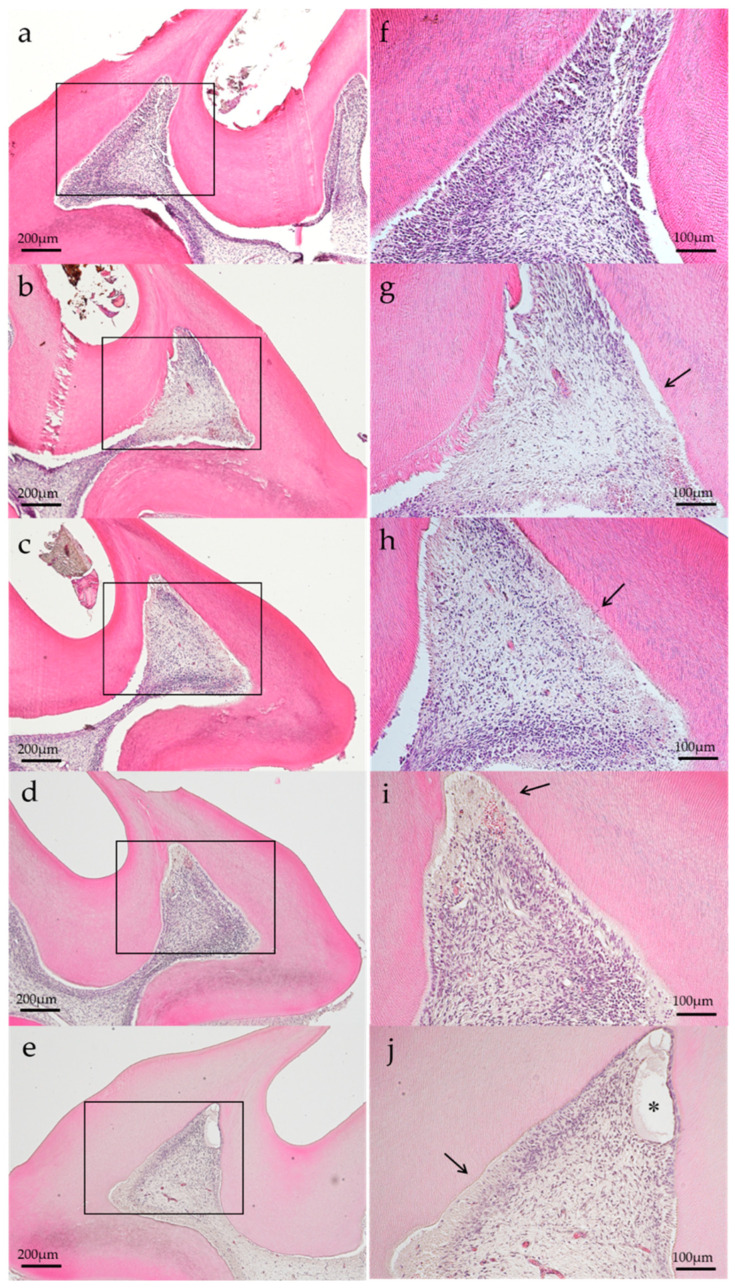
Representative histological images of each group on postoperative day 1. (**a**) Control, (**b**) group 1, (**c**) group 2, (**d**) group 3, and (**e**) group 4. Changes in the odontoblastic layer (arrow) were observed, except for the control specimen. Edematous formation (*) was observed in group 4 specimen. (H–E staining, magnification: (**a**–**e**) 40×, (**f**–**j**): 100×, magnified view of (**a**–**e**), respectively).

**Figure 3:**
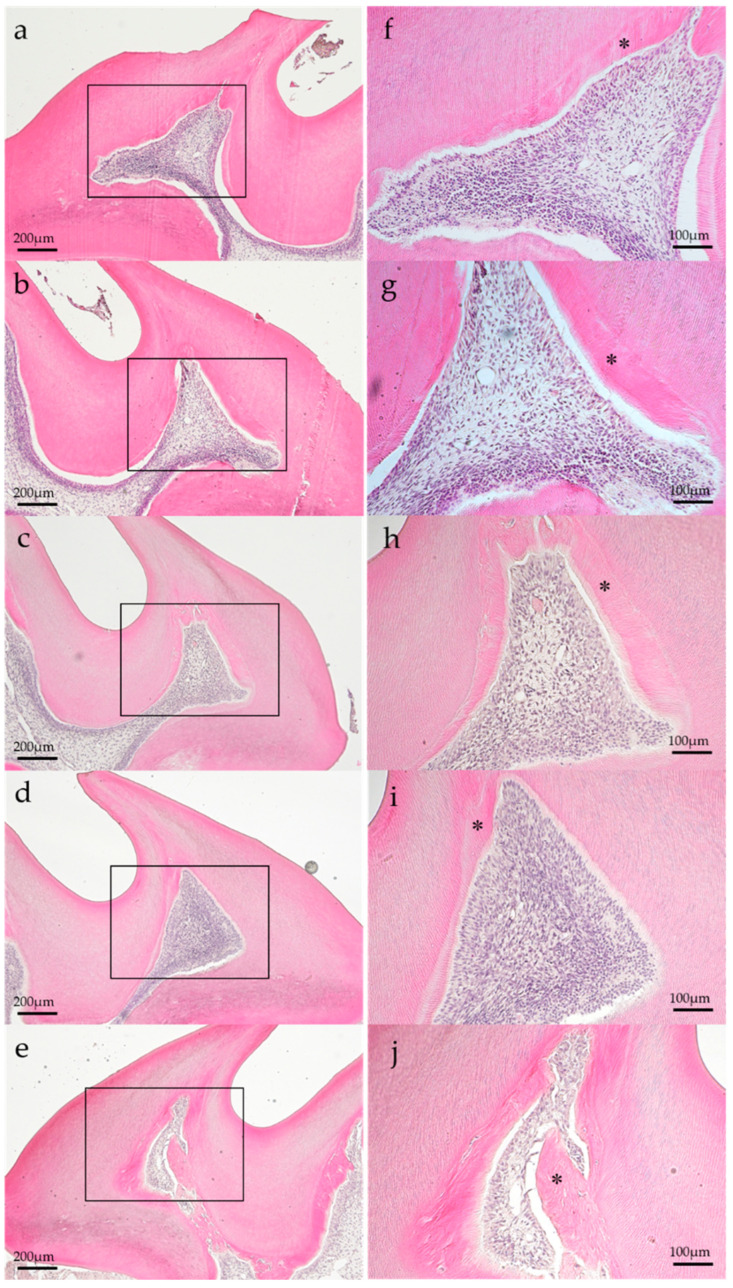
Representative histology images of each group on postoperative day 14. (**a**) Control, (**b**) group 1, (**c**) group 2, (**d**) group 3, and (**e**) group 4. Each group specimen showed normal histology and tertiary dentin formation (*). (H–E staining; magnification, (**a**–**e**) 40×; (**f**–**j**) 100×; magnified view of (**a**–**e**), respectively).

Representative histopathological images of each group are shown in Figure 2 and Figure 3. In the histopathological images taken on postoperatively day 1, the aPDT specimens showed odontoblast loss or decrease in some parts of the odontoblastic layer, and edema formed in the odontoblastic layer in some specimens (Figure 2e). By contrast, the control showed normal pulp tissues. Most specimens showed normal pulp tissue and formed tertiary dentin on postoperative day 14. Specifically, tertiary dentin formation at the base of the pulp floor, away from the cavity, was observed in some specimens (Figure 3e).

### 3.4. Immunohistochemical Observation

**Figure 4:**
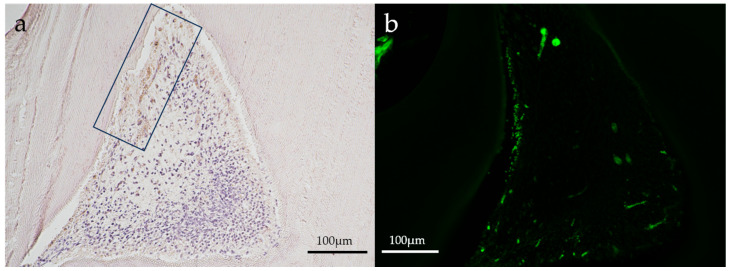
Representative immunohistochemical images of an aPDT specimen (group 2) on postoperative day 1. (**a**) HSP27, (**b**) CD146. A slight positive reaction for HSP27 staining (in a square frame) was seen where the odontoblastic layer might have been destroyed in the aPDT group. CD146 staining (green) was slightly positive in the odontoblastic layer and blood vessels below the odontoblastic layer. (Magnification, 100×).

**Figure 5:**
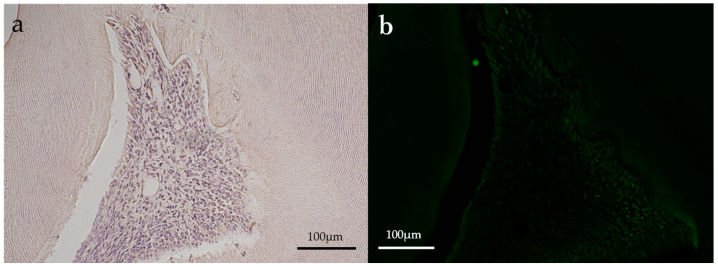
Representative immunohistochemical images of an aPDT specimen (group 2) on postoperative day 14. (**a**) HSP27; (**b**) CD146. Both HSP27 and CD146 staining (green) were almost not observed. (Magnification, 100×).

Representative immunohistochemical images of the aPDT group are shown in Figure 4 and Figure 5.

Slight positive reactions for HSP27 staining were observed in the odontoblastic layer on postoperative day 1 in the aPDT group, whereas the control group showed no positive reaction for HSP27 staining. On postoperative day 14, no positive reactions were observed for HSP27 staining in all experimental groups. CD146 staining was found in the odontoblastic layer and blood vessels below the odontoblastic layer in the aPDT group on postoperative day 1. For CD146 staining on postoperative day 14, the aPDT group showed a slight positive reaction under the tertiary dentin, whereas the control specimen showed no positive reaction.

## 4. Discussion

Most specimens in the aPDT group showed “mild” on the PTD and ICI assessment items on postoperative day 1. The singlet oxygen generated by aPDT and the increased pulp temperature following semiconductor laser irradiation were presumed to be the main factors in pulp irritation.

Singlet oxygen generated by aPDT exhibits a strong oxidative effect, although both the lifetime (0.04 µs) and radiation distance (0.02 µm) are short [18]. Therefore, even if singlet oxygen penetrates the carious dentin close to the dental pulp, it would not pass through the tubules to reach the pulp. In addition, previous studies have reported that aPDT mediated by a microplate or a dentin plate did not reduce the survival rate of cultured cells [19]. From these previous reports and the mild pulp irritation shown in the present study, singlet oxygen unlikely affected the dental pulp in this study. 

This study used tissue-transmitting semiconductor laser, and the laser light irradiated is nearly not absorbed by the tooth substance. After 1 W laser irradiation through a 1 mm dentin plate, the laser power was decreased to 0.216 W; thus, the dentin transmittance of this laser light was approximately 22% [20]. From this report, the laser light used in the aPDT might have penetrated the cavity bottom dentin, and the dental pulp temperature might have risen slightly because of the laser’s exothermic effect, resulting in mild pulpal irritation. Several studies have reported an increase in pulp cavity temperature after applying aPDT. According to Nammour et al., aPDT using phenothiazine chloride as a PS and diode laser (660 nm, 40 mW output) irradiation for 30 s resulted in an average temperature increase in the pulp cavity of 0.83 °C ± 0.22 °C [21]. Mirzaie et al. reported that aPDT using indocyanine green (ICG) as a PS and diode laser (810 nm, 200 mW output) irradiation for 30 s resulted in an average pulp cavity temperature rise of 1.67 °C ± 0.14 °C, and 0.5 W and 1 W diode laser irradiations for 30 s without a PS resulted in an average pulp cavity temperature increase of 4.2 °C and 4.5 °C, respectively [22]. Dental pulp temperature increases beyond approximately 5.5 °C may cause irreversible pulpitis [23]. The pulp cavity temperature increases reported in these previous studies were all <5.5 °C, suggesting that pulp damage caused by aPDT is reversible. Speculatively, the dental pulp reactions observed in the present study were also reversible because the laser power was less than that used in previous studies.

The PTD, ICI, and TDF assessment results on postoperative day 1 showed similarities between group 1, which used MB, and group 2, which used BB. Several studies have reported that MB decreased cell adhesion and proliferation of osteoblasts and fibroblasts and exhibited toxicity when exceeding a certain concentration [24,25,26]; however, no studies have reported BB cytotoxicity. In a previous study, the dentin infiltration depth of MB was 190 µm after a 5 to 30 min contact and shallower than 190 µm for contacts <5 min [27]. Furthermore, MB diluted with water tended to exhibit even lower permeability [28]. In addition, rotary cutting with a diamond point or steel bur creates a smear layer on the surface of the dentin cavity wall, and this smear layer reduces the dentin permeability of PS [29]. These factors were assumed to prevent the PS from penetrating the pulp tissue. Despite the difference in cytotoxicity between MB and BB, the absence of a significant difference between groups 1 and 2 in the histological evaluation might be due to MB not penetrating the pulp cavity. 

The PTD and ICI assessments for the aPDT group were judged as “mild to moderate” on postoperative day 1 and “none” on postoperative day 14, indicating that the pulp irritation caused by aPDT disappeared over time. Although no significant difference in TDF assessment was detected among the aPDT groups, a higher laser power caused greater tertiary dentin formation. More laser light was speculated to penetrate the dentin at higher laser power, increasing the pulp cavity temperature [30]. Consequently, higher-power laser irradiation may have stimulated more pulp cells to form thicker tertiary dentin. In the control group, most specimens had tertiary dentin formation, although the PTD and ICI evaluations were judged as “none.” This could be attributed to the effect of cutting heat during cavity preparation. In group 4, where the laser power was the highest, mild edema was observed in most specimens on postoperative day 1 but not on postoperative day 14; rather, notable tertiary dentin formation was observed. Thus, photobiomodulation therapy (PBMT) may be involved in the healing process of pulp mildly damaged by laser irradiation [31,32]. The mechanism of PBMT is thought to be that light irradiation, such as low-power laser light, alters the activity of cytochrome c oxidase, a photoreceptor molecule localized within the mitochondria, which increases the electrochemical proton gradient, resulting in increased ATP synthesis and cell growth [33].

These results indicate that semiconductor laser irradiation in aPDT causes reversible changes in the pulp through the dentin. Therefore, when aPDT is applied to treat deep carious dentin in live teeth, the laser power and irradiation time must be considered to control the temperature increase in the pulp. aPDT has also been clinically applied to treat periodontal pockets in patients with periodontal disease. Laser light irradiated into periodontal pockets may penetrate the cementum and dentin to reach the pulp. Kreisler et al. measured the temperature in the pulp following laser light irradiation through different dentin thicknesses and revealed that when the dentin thickness was 3 mm, the temperature increase was <3.5 °C, even after continuous irradiation at 2.5 W for 120 s; however, when the dentin thickness was 2 mm, the temperature increase reached 5 °C, which is the critical value for the pulp tissue, after continuous irradiation at 1 W for 20 s and 1.5 W for 10 s [34]. Therefore, because laser irradiation is not performed at such a high power when aPDT is applied, it may be safely used to treat root surface caries without irritating the dental pulp.

A weak positive reaction for HSP27 and CD146 was observed in the odontoblastic layer in aPDT groups on postoperative day 1 but not on postoperative day 14. HSP is a multifunctional protein that appears when generating stresses such as heat and chemicals [35,36]. The positive reaction for HSP27 in the odontoblastic layer may cause initial heat stresses because of laser irradiation. However, the heat stresses may disappear without an HSP27 reaction by postoperative day 14. Several studies have reported that vascular genesis generates tertiary dentin [37,38]. On postoperative day 1, the odontoblastic layer and blood vessels below the odontoblastic layer showed a positive reaction for CD146, suggesting tertiary dentin formation at the CD146 staining position. 

In summary, rat dental pulp was temporarily injured by aPDT but gradually recovered to a normal state with tertiary dentin formation. Thus, the null hypothesis that aPDT would not damage the rat dental pulp in the short term regardless of the PS type or laser irradiation conditions was rejected, but that in the long term was accepted.

## 5. Conclusions

Histopathological evaluation of tooth specimens from rats to which aPDT was applied revealed partial inflammatory changes, mainly in the odontoblast layer, on postoperative day 1, whereas restored-to-normal pulp tissue with the formation of tertiary dentin by postoperative day 14. Although these results are limited due to the use of healthy rat teeth and the limited number of combinations of photosensitizers and semiconductor lasers in aPDT, it is concluded that the application of aPDT caused reversible damage to the rat pulp in the short term, while in the long term, healing occurred with the formation of tertiary dentin.

## Figures and Tables

**Table 1:** Materials used in this study.

Materials	Composition	Lot	Manufacturer
BeautiBond Xtreme	Acetone, purified water, Bis-GMA, carboxylic acid monomers, TEGDMA, phosphate monomers, silane coupling materials, and others	012117	Shofu
Beautifil Flow Plus X F00	Glass powder, Bis-GMA, Bis-MPEPP, TEGDMA, reaction initiators, colorants, and others	072152	Shofu

**Table 2:** Experimental group.

Group	Laser Power/Irradiation Times	PS
Control	Not applicable	Not applicable
Group 1	100 mW/60 s	MB
Group 2	100 mW/60 s	BB
Group 3	50 mW/120 s	BB
Group 4	200 mW/30 s	BB

**Table 3:** Mean (standard deviation) and median (minimum/maximum) values of remaining dentin thickness (µm).

Group	Day 1	Day 14
Control	175.1 (59.1), 170.3 (101/257)	205.7 (54.5), 197.9 (133/310)
Group 1	218.3 (76.2), 220.3 (131/346)	234.7 (39.5), 240.9 (184/277)
Group 2	180.6 (39.6), 170.1 (150/260)	198.1 (63.4), 197.2 (124/264)
Group 3	239.0 (23.5), 244.5 (205/268)	242.9 (19.0), 247.0 (216/264)
Group 4	240.3 (19.5), 238.3 (210/266)	251.9 (15.2), 253.6 (231/267)

## Data Availability

The data presented in this study are available on request from the corresponding author.
